# Exploration of advanced cellulosic material for membrane filtration with outstanding antifouling property

**DOI:** 10.1039/d2ra08165b

**Published:** 2023-03-08

**Authors:** Hiroshi Koyama, Taro Mori, Kanji Nagai, Shu Shimamoto

**Affiliations:** a Business Development Center, Innovation and Business Development Headquarters, Daicel Corporation Japan; b Graduate School of Natural Science and Technology, Kanazawa University Japan; c Biomass Innovation Center, Daicel Corporation Japan; d Life Sciences R&D Center, CPI Company, Daicel Corporation Japan

## Abstract

Membranes, at times, have issues due to membrane fouling. The membrane fouling leads to performance deterioration and poses a potential to clog the membrane. Here we present experimental works carried out with emphasis on the antifouling properties, chlorine resistance, and mechanical properties of cellulose triacetate (CTA) and cellulose esters. We present that antifouling performance of cellulose esters evaluated by means of the VCG theory decreases with increasing carbon number in the substituent because of the high electron-donating nature of short aliphatic ester groups. When a long aliphatic ester group is required in terms of other properties such as resistance to chlorine, introducing it together with another substituent with an electron-donating nature such as an ethylene glycol moiety may strike a balance between antifouling and other performances.

## Abstract 2

We revealed that a high electron donor ability (*γ*^−^) is the origin of the excellent antifouling properties of cellulose triacetate (CTA); with a view to further improving antifouling properties of CTA, molecular designs to increase *γ*^−^ are required.

## Graphical abstract

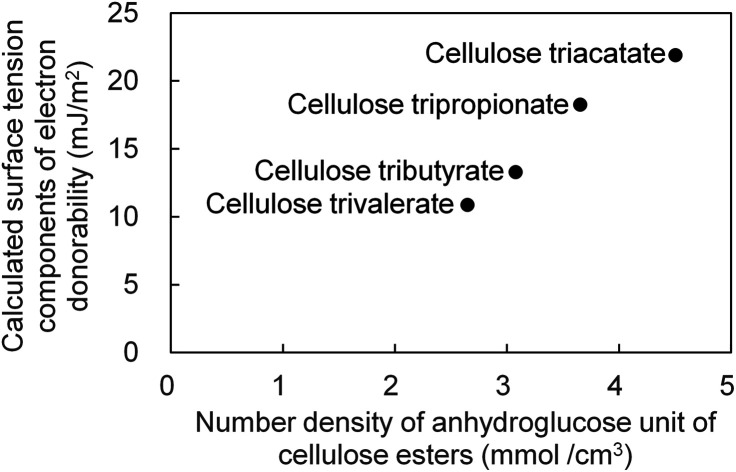

## 1 Introduction

In recent years, the demand for new water resources has increased worldwide. Statistical projections suggest that approximately 25% of the population will face water scarcity by 2030.^1^ Water scarcity has increased significantly owing to exponential population growth, continued industrialization, increased agricultural activity, water pollution, mismanagement of water resources, and climate change.^2,3^ Goal 6 of the United Nations sustainable development goals (SDGs) is clean water and sanitation.

However, to produce daily drinking water from surface water, such as seawater and river water, it is necessary to eliminate suspended matter and microorganisms present in raw water. Methods such as sand filtration,^4^ coagulation sedimentation,^5^ distillation,^6^ and membrane filtration^7^ are commonly used to eliminate impurities from raw water. Membrane filtration is one of the most important and effective methods for solving global water shortage problems, and hence, has expanded into areas of material recovery,^8^ wastewater treatment,^9^ water supply^10^ and sewage.^11^ If the raw water contains impurities during membrane filtration, the membrane is clogged or cake layers are formed on the membrane surface, which significantly decreases the permeation performance. This phenomenon is called “fouling”^12^ and hinders efficient operation. Substances that cause fouling include polysaccharides,^13^ proteins,^14^ bacteria,^15^ and viruses^16^ (organic substances derived from living organisms), and soil and clay (inorganic substances derived from minerals), which are mixed with raw water as fine particles (turbidity)^14^ and solutes.^17^ Therefore, for efficient membrane filtration, it is necessary to prevent or reduce fouling.

Physical methods such as cross-flow filtration^18^ and backwashing,^14^ and chemical methods such as using acids, alkalis, surfactants, and oxidizing agents,^19^ have been used to reduce fouling. In general, materials that interact less with foulants as a membrane material are used to easily remove foulants, and methods such as modification of the surface of the membrane^20^ are used to reduce interactions with foulants. At times, inorganic materials such as carbon nanotubes are added to the membrane.^21^ They are often used in combination with various techniques depending on the type and amount of foulant in the feed raw water and the type of filtering medium.

Some of the membrane materials used in membrane filtration are cellulose acetate (CA),^22^ polyethersulfone (PES),^23^ polyamide (PA),^24^ polyacrylonitrile (PAN),^25^ and polyvinylidene fluoride (PVDF).^26^ However, most commercially available membranes are prepared from hydrophobic materials, which tend to adsorb or deposit on the surface or pores of the membrane, thereby reducing the filtration rate.^27^

Therefore, many studies have been conducted to modify the polymer of membrane materials. As fouling is first caused by the interaction between the foulant and the membrane,^28^ many improvement measures focusing on surface modification have been investigated, such as grafting,^29,30^ coating,^31^ interfacial polymerization,^28^ use of artificial nanomaterials,^21^ surface patterning,^32^ and introduction of a conductive layer.^33^

CA is a good membrane material with excellent antifouling properties.^34^ Currently, cellulose triacetate (CTA, degree of substitution (DS) = 2.9) and cellulose diacetate (CDA, DS = 2.5) are the most widely used cellulose acetates in industries. In general, CDA is more hydrophilic, more soluble in a wider range of solvents,^35,36^ and more biodegradable^37^ compared to CTA. The first CA used in polymer membranes for water treatment was CDA,^38^ considering it is easy to control the porous structure of the membrane owing to its high solvent solubility, and the water permeability of the resulting polymer membrane is excellent because of its high hydrophilicity. However, owing to its high biodegradability, CDA is unsuitable for long-term use in water treatment applications.^37^ Therefore, CTA is currently used.

Sodium hypochlorite has been used for a long time because it is effective in maintaining the high permeability of CTA and other membrane materials and can maintain the sterilizing effect of water supplies. Sodium hypochlorite acts as an oxidizing agent to generate highly polar oxygen-containing functional groups on the foulant surface, where polar functional groups reduce the hydrophobic interactions between the foulant and the polymeric membrane material. Therefore, sodium hypochlorite facilitates the removal of foulants such as polysaccharides,^13^ proteins,^14^ and bacteria^15^ deposited on the surface of polymer membranes during water treatment.

Sodium hypochlorite is also used to prevent the growth of microorganisms on the surface of CA membranes and the biodegradation of CA.^39,40^ For these purposes, sodium hypochlorite is required to clean CA membranes and inhibit biodegradation. However, when CA membranes are exposed to sodium hypochlorite for a long time, and the oxidation reaction by hypochlorite causes CA main-chain scission, membrane embrittlement, and easy destruction of the membrane.^41^

Additionally, sodium hypochlorite treatment of polymer membranes for desalination of seawater lowers sodium chloride removal performance.^39,42,43^ To improve the fouling problem, much research has been conducted on the types of foulants that reduce the permeation rate of membranes. In the 1990s, hydrophobic organic matter was identified as the main cause of membrane fouling.^44^ Since 2000, an increasing number of studies have confirmed that hydrophilic organic matter causes more serious fouling.^45^ Therefore, to reduce fouling, it is very important to evaluate the strength of the interaction between the membrane and the foulant. One method for this is van Oss–Chaudhury–Good theory (VCG theory).^46,47^ According to this method, antifouling properties can be estimated from the contact angles of the membrane material and foulants.

Cornelissen *et al.*^48^ analyzed the interactions between various membrane polymers and human serum albumin (HSA), and polyethylene glycol (PEG). The authors verified that interfacial free energy between membrane material 1 and foulant 2 in aqueous media 3 (Δ*G*_132_) is a measure of fouling tendency; Δ*G*_132_ between materials know as low fouling tendency such as cellulose acetate, and model foulant such as HSA is high while that with materials known as high fouling tendency is low. The authors while admitting the concept of applying the VCG theory to the fouling problem neglects the effects of pore size and pore size distributions which do have a large effect on membrane fouling in practice concluded that, apart from aforementioned shortcoming, the concept of the VCG theory is a powerful tool in predicting the adsorptive fouling tendency of ultrafiltration and microfiltration membranes. Białopiotrowicz *et al.*^49^ analyzed the interaction between CA and bovine serum albumin (BSA). Subhi *et al.*^50^ analyzed the interaction of polyvinylidene fluoride with BSA, humic acid (HA), and sodium alginate (SA). Meng *et al.*^51^ evaluated the differences in fouling behavior depending on the polysaccharide structure. Attempts have also been made to investigate the interactions between the membranes and the foulants using computational chemistry techniques.^52^ Including the above example, although several studies have been conducted on the interaction of CA with foulants, there are few studies on the interactions between cellulose derivatives, except CA and foulants.^53^

This study investigated the interactions between foulants and cellulose esters, which have systematically changed acyl groups. Additionally, as described above, CA deteriorates by washing with sodium hypochlorite water, which is performed to restore the permeation flow rate decreased by fouling. Therefore, we investigated the effects of cellulose substituents on these problems.

## 2 Experimental part

### 2.1 Materials

Cellulose triacetate (a) [CTA(a)], cellulose tripropionate (CTP), cellulose tributyrate (CTB), and cellulose trivalerate (CTV) were synthesized using Ceolus PH-101 (Asahi Kasei) as cellulose. Lyocell fibers (Tencel, Lenzing)^54^ cut into 5 mm lengths were used as cellulose for the synthesis of cellulose laurate and cellulose laurate trioxadecanoate (a) and (b). Cellulose triacetate (b) used was LT-75 (Daicel). Sumika Excel 3600P (Sumitomo Chemical) was used as polyethersulfone (PES). 4-Dimethylaminopyridine (99%), lithium chloride (99%), acetyl chloride (98%), thionyl chloride (99%), chloroform (for HPLC), tetrahydrofuran (99.5%), diiodomethane (97%), formamide (98.5%), and *N*,*N*-dimethylacetamide (ultra-hydrated) were purchased from Fujifilm Wako Pure Chemical Co. Propionyl chloride (98%), butyryl chloride (98%), valeryl chloride (98%) and [2-(2-methoxyethoxy)ethoxy]acetic acid (95%) were purchased from Tokyo Kasei Kogyo. Unless otherwise stated, the raw materials and solvents were used as received without further purification.

### 2.2 Synthesis of cellulose esters

**Scheme 1:**
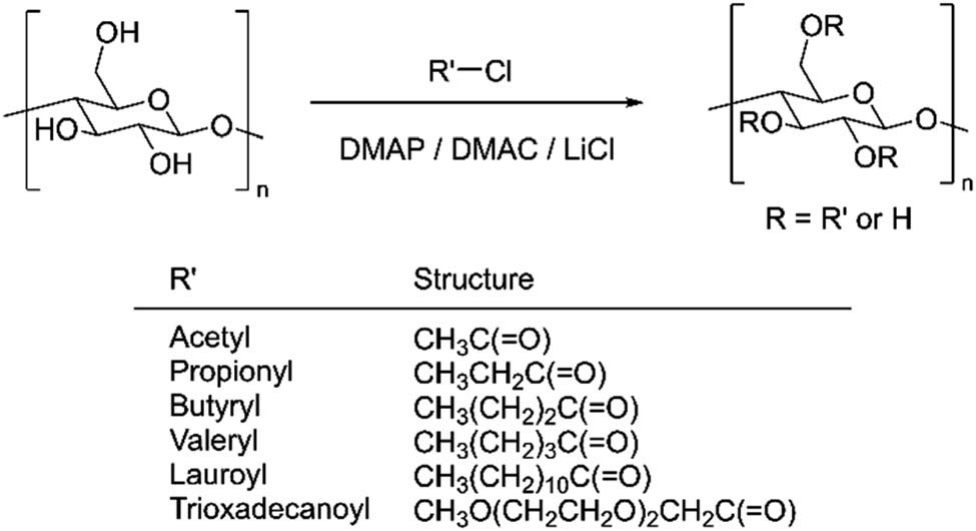
Esterification of cellulose using acid chloride.

The reaction is shown in Scheme 1. 0.405 g (2.5 mmol, as anhydrous glucose unit) of cellulose (Tencel or Ceolus PH-101) was weighed into a flask and 14.0 g of dehydrated dimethylacetamide (DMAC) was added under nitrogen flow. The temperature was increased to 130 °C and the mixture was stirred for 2 h. Then, the mixture was cooled to room temperature, and 1.01 g of lithium chloride (LiCl) was added. The temperature was increased to 150 °C at a rate of 20 °C/10 min. After the temperature of the solution reached 150 °C, the mixture was heated and stirred while maintaining the temperature at 150 °C for 20 to 30 min, and slowly cooled to room temperature. After the cellulose was completely dissolved in the solution, 0.90 g (7.4 mmol) of 4-dimethylaminopyridine (DMAP) was added and the temperature was raised to 80 °C. After DMAP was completely dissolved, 22.5 mmol of the acid chloride corresponding to the target substance was added dropwise and reacted at 80 °C for 3 h. When the reaction was complete, methanol was added dropwise and stirred for approximately 30 min to quench the unreacted acyl chloride reagent, and a large amount of methanol was added to precipitate the product, which was filtered and washed with methanol. The product was dissolved in THF and re-precipitated by the addition of methanol. The obtained solid was collected by filtration, washed with methanol, and vacuum dried at 40 °C to constant weight. Subsequently, the target compound was obtained.

Additionally, 11.25 mmol of lauroyl chloride and 11.25 mmol of trioxadecanoyl chloride were used as acyl chlorides for synthesizing CLTOD(a), and 7.5 mmol of lauroyl chloride and 15.0 mmol of trioxadecanoyl chloride were used for synthesizing CLTOD(b). Table 1 summarizes the analytical results for each cellulose ester synthesized in this study.

**Table 1:** DS, *M*_n_, *M*_w_, DPw, molecular mass of repeat unit (*M*_R_) of cellulose esters

Cellulose esters		DS				*M*_n_ (g mol^−1^)	*M*_w_ (g mol^−1^)	DPw	*M*_R_ a (g mol^−1^)
		Substituent 1		Substituent 2					
CTA(a)	Cellulose triacetate	Acetyl	3.0	—		12 400	65 500	230	288.2
CTP	Cellulose tripropionate	Propionyl	2.9	—		29 900	79 600	240	330.3
CTB	Cellulose tributyrate	Butyryl	3.1	—		36 100	92 600	250	372.4
CTV	Cellulose trivalerate	Valeryl	3.1	—		34 600	85 300	210	414.5
CTA(b)	Cellulose triacetate	Acetyl	2.9	—		94 000	289 000	1000	288.2
CTL	Cellulose trilaurate	Lauroyl	3.0	—		73 000	232 000	330	709.0
CLTOD(a)	Cellulose laurate trioxadecanoate	Lauroyl	1.7	Trioxadecanoyl	1.3	64 700	255 000	370	680.3
CLTOD(b)	Cellulose laurate trioxadecanoate	Lauroyl	1.1	Trioxadecanoyl	1.9	73 900	258 000	390	667.0

a The molecular mass of the repeat unit was calculated as DS 3.0.

### 2.3 Synthesis of trioxadecanoyl chloride

[2-(2-Methoxyethoxy) ethoxy] acetic acid (20.00 g, 0.11 mol) was added to a 100 mL three-necked flask, and thionyl chloride (14.69 g, 0.12 mol) was added dropwise over 20 min with stirring at room temperature under nitrogen flow. Subsequently, the temperature was gradually increased, and the reaction solution was stirred at 40–50 °C until the generation of bubbles ceased. Furthermore, the temperature was increased to 60 °C, and the mixture was stirred for 1 h. After cooling to room temperature, excess thionyl chloride was distilled off to obtain the target compound (21.43 g, 0.11 mol, yield 97.1%).

### 2.4 Determination of degree of substitution (DS)

10 mg of cellulose derivative was dissolved in 1 g of chloroform-d, and ^1^H NMR measurement was performed at room temperature using JEOL JNM-ECA 500 spectrometers. DS was calculated from the area ratio of the protons of the terminal methyl group or terminal methoxy group of the substituent and the protons of the cellulose backbone.

### 2.5 Contact angle measurement

The film used for the contact angle measurements was prepared by coating a glass plate with a dope obtained by dissolving cellulose derivatives at a concentration of 5% in dichloromethane and drying at ambient temperature and pressure. The PES film was prepared by dissolving PES in DMAC at a concentration of 5%, coating the dope on a glass plate, and drying it at 100 °C. The contact angle measurements were performed at 20 °C using a DropMaster 700 manufactured by Kyowa Interface Science Co., Ltd., with a film formed on glass as a sample.

**Fig. 1:**
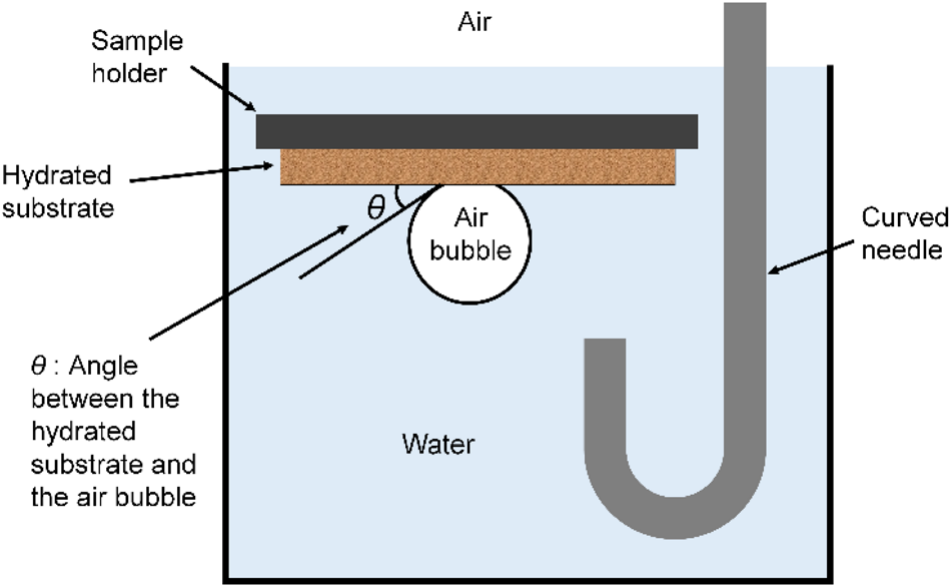
Captive bubble method.

The sessile drop method uses a dried film, drops liquid from a syringe onto the film, and measures the contact angle of the droplet. In the captive bubble method, a film that had been dried and immersed in distilled water for one day was used. The air bubble was brought into contact with the film, and the contact angle between the hydrated substrate and the air bubble was measured as shown in Fig. 1.

Statistical analyses (Tukey Kramer tests) were carried out for contact angle results.

### 2.6 Tensile test

The test piece used for the tensile test was formed by casting 0.1 g mL^−1^ concentration of a cellulose derivative in dichloromethane solution on a glass plate, air-drying and peeling it off from the glass, and punching it into a dumbbell-shaped test piece. After completely removing the solvent by immersing the test pieces in water for at least 6 h, the water droplets on the surface were lightly wiped off and used in a wet state. The test pieces of PES were prepared by casting a solution of 10 wt% PES in DMAC on a glass plate, drying it by heating at 120 °C, punched into dumbbell-shaped test pieces, and processing them in the same manner as for cellulose derivative films. The tensile test was performed using a Shimadzu EZ-Test (EZ-SX).

### 2.7 Chlorine resistance test

The chlorine resistance test was performed according to Hashizume *et al.*'s method.^41^ The test piece used for the tensile test was formed by casting 0.1 g mL^−1^ concentration of a cellulose derivative in dichloromethane solution on a glass plate, air-drying and peeling it off from the glass. The resulting film was processed and prepared in the same manner as in the tensile test to obtain the test pieces. Then, a tensile test was performed after the test piece was immersed in an aqueous solution of sodium hypochlorite with an effective chlorine concentration of 20 000 ppm, and the time required for tensile elongation at break to reach 90% of the initial value was measured. This time was used as an index of chlorine resistance. A hypochlorite concentration meter (Handy water quality meter “Aquab” AQ-202 manufactured by Shibata Science Co., Ltd.) was used to measure the effective chlorine concentration.

### 2.8 Calculation of the number density of anhydroglucose units in cellulose esters

The number density of anhydroglucose units in the cellulose ester was obtained by dividing the formula weight (*M*_R_) of the anhydroglucose units of each cellulose ester by the density of each cellulose ester. The formula weights of the anhydroglucose units were calculated assuming the DS of all cellulose esters was 3.0. The values of Malm *et al.*^55^ were used to calculate the densities of the CTA, CTP, and CTB. The density of the CTV was determined using the method proposed by Malm *et al.*^55^ as follows.

First, the volume of the cellulose portion was determined. The formula weight of the anhydrocellulose residue of cellulose, 162, was divided by the density of cellulose, 1.52, and the total volume of cellulose was calculated as 106.6 cm^3^ mol^−1^. From this, the volume of three hydrogen atoms (5.5 cm^3^ mol^−1^ × 3) was subtracted to calculate the volume of cellulose with no hydroxyl hydrogen as:106.6 − 5.5 × 3 = 90.1 cm^3^ mol^−1^

Subsequently, the volume of the valeryl group was determined. The volume of valeric acid, 108.88 cm^3^ mol^−1^, was calculated by dividing the formula weight of valeric acid, 102.13 g mol^−1^, by its density (0.938288 g cm^−3^).^56^ The volume of the valeryl group is the value obtained by subtracting the volume of the oxygen atom (7.8 cm^3^ mol^−1^) and the hydrogen atom (5.5 cm^3^ mol^−1^) from that of the volume of valeric acid as:108.88 − 7.8 − 5.5 = 95.58 cm^3^ mol^−1^

As the volume of CTV is the sum of the volume of the cellulose portion and the volume of the three valeryl groups, it was calculated as:90.1 + 95.58 × 3 = 376.84 g cm^−3^

### 2.9 Molecular weight measurement

The molecular weight of each cellulose ester was measured using a Prominence GPC system (manufactured by Shimadzu Corporation) as the apparatus, SHODEX RI-101 (manufactured by Showa Denko) as an RI detector, Tskgel (7.8 mm × 30 cm, manufactured by Tosoh) as a column, and chloroform as the mobile phase. GPC measurements were performed at an oven temperature of 40 °C. Polystyrene was used as the standard.

## 3 Result and discussion

### 3.1 Analysis of interfacial tension based on van Oss–Chaudhury–Good theory (VCG theory)

The surface energy of the materials used in this study was evaluated by using the method (VCG theory) proposed by van Oss *et al.*^46,47^ This method is based on the assumption that the surface tensions of liquids and solids can be expressed as the sum of nonpolar forces (Lifshitz–van der Waals (LW) forces) (*γ*^LW^), polar (Lewis acid–base (AB) interaction) forces (*γ*^AB^), electrostatic interactions (*γ*^EL^), and interaction forces due to Brownian movement (*γ*^BR^).

Among them, the polar (Lewis acid–base (AB) interaction) force (*γ*^AB^) comprises the electron-acceptor component *γ*^+^ and electron-donor component *γ*^−^, and *γ*^AB^ is expressed by the geometric mean of the electron-acceptor and electron-donor components.1
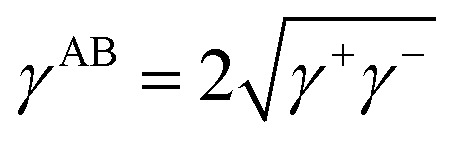
Here, the contribution of the electrostatic interaction and the force of Brownian motion is considered to be smaller than the contribution of the nonpolar and polar interactions.^48^ Therefore, the total surface tension can be expressed as the sum of *γ*^LW^ and *γ*^AB^: in other words, the surface tension *γ* can be expressed by surface tension components, such as *γ*^LW^, *γ*^+^, and *γ*^−^.2
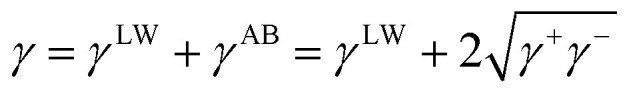


**Fig. 2:**
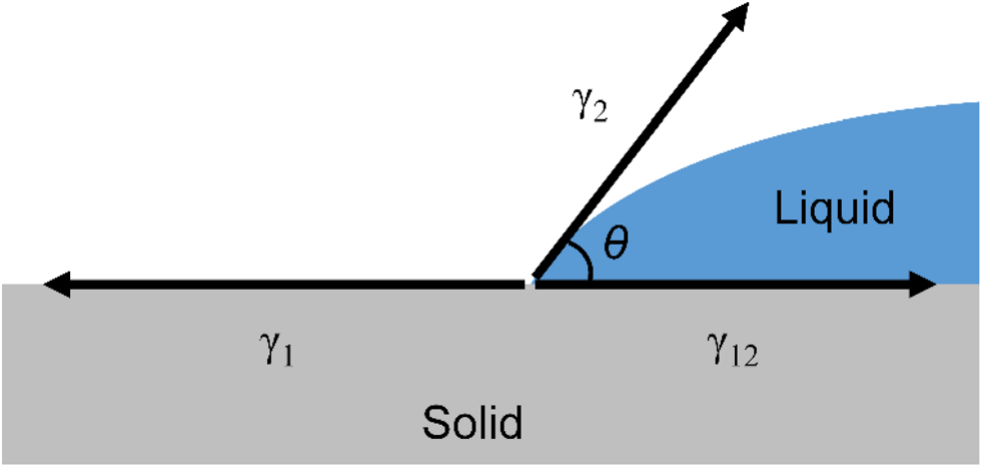
The Young equation.

The relationship between the solid–liquid interfacial tension (*γ*_12_), solid surface tension (*γ*_1_), liquid surface tension (*γ*_2_), and contact angle (*θ*) of a droplet on a solid surface is expressed by Young's equation, as shown in Fig. 2. (Solids are denoted by subscript 1 and liquids by subscript 2.)

3 *γ*_1_ = *γ*_12_ + *γ*_2_ cos *θ*

From Dupre's equation, the free energy Δ*G*_12_ of the solid–liquid interface interaction is expressed as (1 represents solid, 2 represents liquid):

4 Δ*G*_12_ = *γ*_12_ − *γ*_1_ − *γ*_2_

Furthermore, the free energy of the solid–liquid interface interaction in the state immersed in the third liquid can be expressed as (the third liquid is represented by the subscript 3).

5 Δ*G*_132_ = *γ*_12_ − *γ*_13_ − *γ*_23_

The following equation is derived from eqn (3) and (5).6
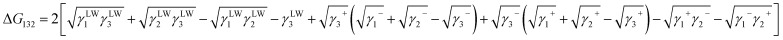


The free energy of the interfacial interaction can be calculated by substituting the value of each surface tension component into the above equation. If the free energy of this interfacial interaction (Δ*G*_132_) is positive, there is no interaction between the solid and the liquid. If it is negative, the larger its absolute value, the stronger is the solid and liquid adhesion. Therefore, this value (Δ*G*_132_) can be used as an index of the antifouling properties of water treatment membranes.

Next, the method to obtain the surface tension component will be described. From eqn (3) and (4), the following Young–Dupre equation is derived:

7 Δ*G*_12_ = −*γ*_1_(1 + cos *θ*)

The following equation is derived from eqn (2) and (4):8
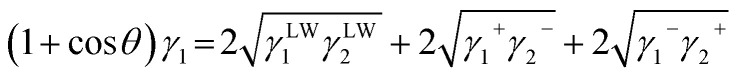


If we measure the contact angle with a solid using three solvents with known interfacial tensions of *γ*^LW^, *γ*^+^, and *γ*^−^, three equations are created. Each surface tension component of the solid can be calculated by solving these three simultaneous equations, each surface tension component of the solid can be calculated.

Additionally, because the surface properties of membranes and foulants change at different pH values, it is necessary to use the contact angle and surface tension components of the membranes and foulants at the pH of the operating conditions. In this study, the contact angle and surface tension were at pH 7.

In the next section, we discuss the surface tension components of cellulose triesters with systematically changed substituent carbon numbers.

### 3.2 Effect of the carbon number of substituents on cellulose esters

Table 2 shows the results of measuring the contact angles of the cellulose triesters and solvents. Cellulose esters with acyl groups and carbon numbers ranging from 2–5 were used, and water, formamide, and diiodomethane were used as solvents. For any solvent, the contact angle increased as the number of carbon atoms in the substituent increased, thereby indicating that the affinity for each solvent decreased as the number of carbon atoms in the substituent increased. Table 3 shows the surface tension components of each solvent used for calculating the surface tension components of cellulose triester in this study. Table 4 shows the results of the calculation of the surface tension component of each cellulose triester based on the values in Tables 2 and 3.

**Table 2:** Average figures^a^, standard deviation, and number of measurements of the measured contact angles of formamide, diiodomethane, and water on cellulose esters with different carbon numbers in the substituent

Cellulose esters	Number of carbon atoms in the substituent	Formamide			Diiodomethane			Water		
		Average (deg)	SD (deg)	*n*	Average (deg)	SD (deg)	*n*	Average (deg)	SD(deg)	*n*
CTA(a)	2	50.7^a^	1.8	28	29.0^a^	1.2	30	61.7^a^	0.9	30
CTP	3	61.3^b^	1.1	29	35.8^b^	0.9	30	70.3^b^	1.0	38
CTB	4	71.2^c^	0.6	29	41.0^c^	0.6	30	80.2^c^	0.8	38
CTV	5	78.7^d^	0.8	30	45.2^d^	0.6	30	86.9^d^	0.9	39

a Values in the same column with different letters are significantly different (*p* < 0.05).

**Table 3:** Surface tension components (mJ m^−2^) of test liquid^46^

Liquid	*γ* ^LW^ (mJ m^−2^)	*γ* ^+^ (mJ m^−2^)	*γ* ^−^ (mJ m^−2^)
Water	21.8	25.5	25.5
Formamide	39.0	2.3	39.6
Diiodomethane	50.8	0.0	0.0

**Table 4:** Calculated surface tension components of cellulose esters with different carbon number in the substituent

Cellulose esters	Number of carbon atoms in the substituent	*γ* ^LW^ (mJ m^−2^)	*γ* ^+^ (mJ m^−2^)	*γ* ^−^ (mJ m^−2^)
CTA(a)	2	44.6	0.05	21.9
CTP	3	41.7	0.4	18.2
CTB	4	39.1	1.0	13.3
CTV	5	36.9	1.7	10.9

**Fig. 3:**
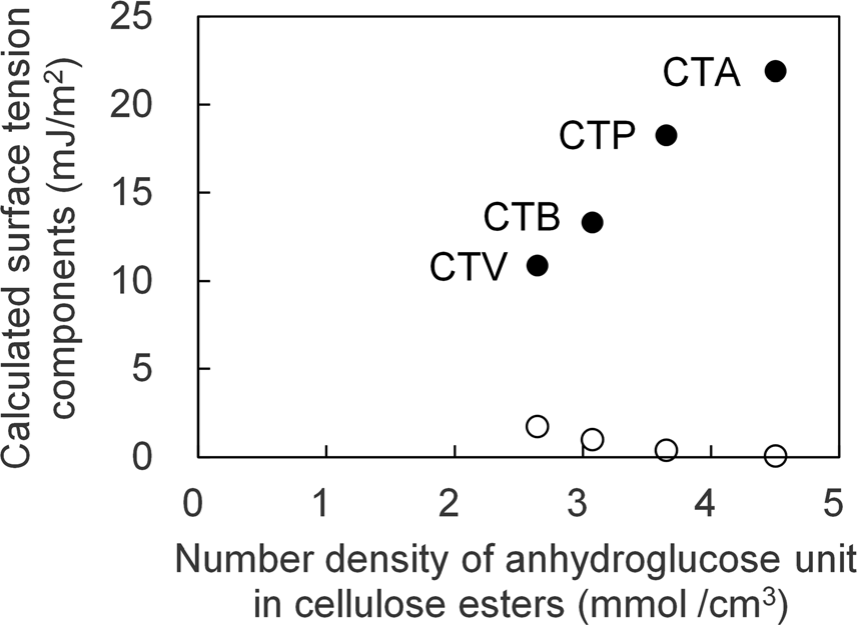
Plot of calculated surface tension components (*γ*^+^: open circles, *γ*^−^: filled circles) *versus* number density of anhydroglucose unit in cellulose esters with different carbon number in substituent.

As shown in Table 4, when the number of carbon atoms in the substituent of the cellulose triester increased from 2 to 5, *γ*^LW^ decreased by approximately 17%, but *γ*^−^ decreased by approximately 50%, and *γ*^+^ increased by 34 times. The differences in *γ*^−^ and *γ*^+^ was large in these cellulose esters. The reason for the decrease in *γ*^−^ is that the larger substituents increased the volume per anhydrocellulose residue and further number density of the anhydroglucose residues of the cellulose triesters. Because *γ*^−^ is linearly related to the number density of diluted the surface tension component. Therefore, *γ*^+^ and *γ*^−^ of the cellulose triester are shown in Fig. 3 as a function of the anhydroglucose residues in cellulose triesters, the change in *γ*^−^ as the number of substituent carbon atoms increases is considered to be largely due to the dilution effect. However, *γ*^+^ increased even when the number density of the anhydroglucose residues decreased, thereby indicating that the change in *γ*^+^ was caused by another factor. However, the mechanism of this phenomenon requires further investigation.

Yuan and Zydney^44^ investigated the fouling phenomenon of humic acid (HA), which is a foulant in river water. Cornelissen *et al.*^48^ studied HSA and PEG and Białopiotrowicz *et al.*^49^ studied BSA as a model substance for foulants. In this study, we evaluated the free energy of the interfacial interaction between cellulose triester and foulants or the foulant model substances mentioned above. The surface tension components of these substances are listed in Table 5. The free energies of the polymer-foulant interfacial interactions were calculated using eqn (6) with these values. The results are shown in Fig. 3. The more positive Δ*G*_132_ is, the less the foulant adheres, and *vice versa*. When the foulants are the same and the substituents of the cellulose ester are changed, as the number of carbon atoms in the substituents increases, Δ*G*_132_ becomes more negative, and the foulants adhere more easily. When the substituents of the cellulose ester were the same and the foulants were changed, Δ*G*_132_ of cellulose ester and HSA, PEG, and BSA were similar; however, Δ*G*_132_ of cellulose ester and HA was negative compared with Δ*G*_132_ of other foulants. Therefore, HA tends to adhere to these cellulose esters more easily. To understand this tendency, the calculated Δ*G*_132_ was analyzed in detail. The right-hand side of eqn (6), which defines Δ*G*_132_, can be divided into the following four terms:i
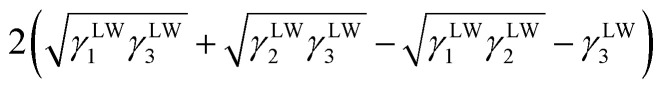
ii
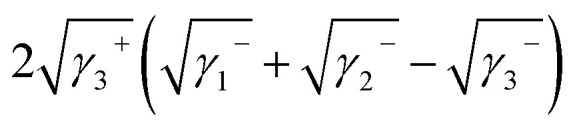
iii
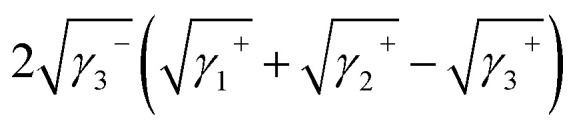
iv
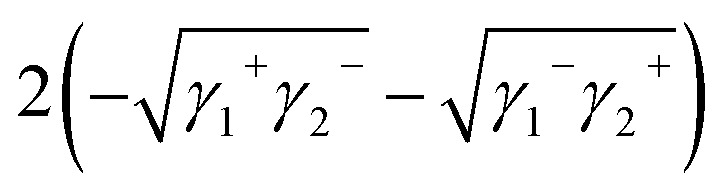


**Table 5:** Surface tension components (mJ m^−2^) of model foulants (HSA, BSA, PEG) and potential foulants (HA)

Foulants	*γ* ^LW^ (mJ m^−2^)	*γ* ^+^ (mJ m^−2^)	*γ* ^−^ (mJ m^−2^)	Ref.
Human serum albumin (HSA)^a^	26.8	6.3	50.6	46
Bovine serum albumin (BSA)^b^	28.9	0.0	63.6	49
Polyethylene glycol (PEG)	43.0	0.0	64.0	46
Humic acid (HA)^c^	30.8	3.6	12.7	57

a Hydrated, two layers of hydration water, pH 7.

b Surface tension components of hydrated bovine serum albumin.

c pH 7.

Table 6 shows the breakdown of Δ*G*_132_, as shown in Fig. 3 into items (i)–(iv). The results are as follows. The value of term (i), which is related to *γ*^LW^, is small in all cases. Term (ii) has a large positive value because the *γ*^−^ values of the cellulose triesters and foulants are large. CTV, which is a cellulose triester with a large number of substituent carbon atoms, and HA have a small value for this term because their *γ*^−^ is small. Term (iii) has a negative value because the *γ*^+^ of the membrane materials and foulants is smaller than *γ*^+^ of water. The value of term (iv) is small or negative in the combination of CTV and any foulant or HA and any cellulose ester owing to the large *γ*^+^ of CTV or HA.

**Table 6:** Breakdown of the calculated interfacial interaction energies (Δ*G*_132_) between foulants (HSA, PEG, BSA, HA) and cellulose esters with different carbon number in substituent

Components of Δ*G*_132_		Foulants	CTA(a) (mJ m^−2^)	CTP (mJ m^−2^)	CTB (mJ m^−2^)	CTV (mJ m^−2^)
(i)	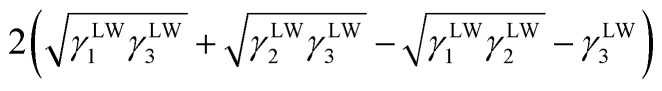	HSA	−2	−2	−2	−1
		PEG	−8	−7	−6	−5
		BSA	−3	−3	−2	−2
		HA	−4	−3	−3	−2
(ii)	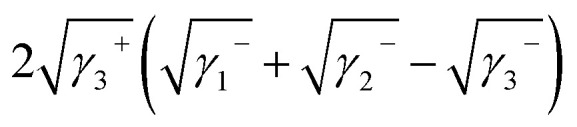	HSA	68	64	58	54
		PEG	77	73	67	63
		BSA	77	73	66	63
		HA	32	28	22	18
(iii)	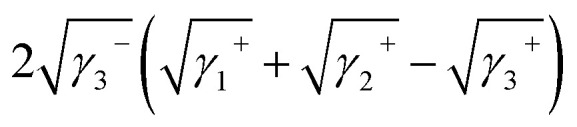	HSA	−23	−20	−16	−12
		PEG	−49	−45	−41	−38
		BSA	−49	−45	−41	−38
		HA	−30	−26	−22	−19
(iv)	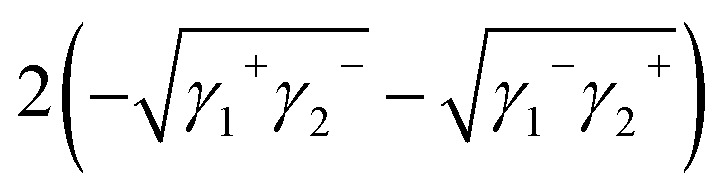	HSA	−27	−30	−32	−35
		PEG	−4	−10	−16	−21
		BSA	−4	−10	−16	−21
		HA	−19	−21	−21	−22
Δ*G*_132_	(i) + (ii) + (iii) + (iv)	HSA	16	13	8	5
		PEG	17	12	4	−1
		BSA	22	16	7	2
		HA	−20	−21	−24	−25

**Fig. 4:**
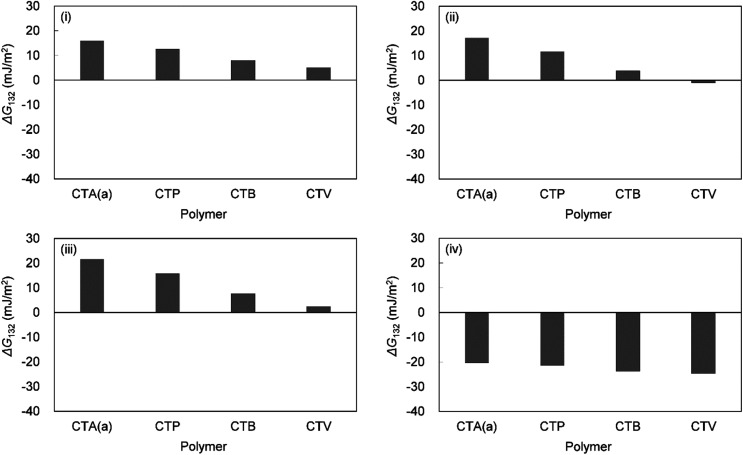
Calculated interaction energies between foulants ((i) HSA, (ii) PEG, (iii) BSA, (iv) HA) and cellulose esters with different carbon number in substituent in water (Δ*G*_132_).

To summarize the above, Δ*G*_132_ decreases (antifouling property decreases similarly) as the carbon number of the substituents on the cellulose triester increases, considering as the number of substituent carbon atoms on cellulose esters increases, the *γ*^−^ of cellulose esters decreases and *γ*^+^ of them increases, and hence, terms (ii) and (iv) decrease. Δ*G*_132_ between HA and each cellulose triester is negative because terms (ii) and (iv) become more negative owing to the small *γ*^−^ and large *γ*^+^ of HA (Fig. 4).

Based on these results, for a foulant with large *γ*^−^ and small *γ*^+^, selecting a cellulose triester with large *γ*^−^ and small *γ*^+^ increases Δ*G*_132_ and improves the antifouling property within the range of cellulose triesters and the foulants examined in this study. Furthermore, *γ*^LW^ had little effect.

Therefore, CTA with a large *γ*^−^ and a small *γ*^+^ has the best antifouling properties among these cellulose triesters, and the longer the acyl group, the lower the antifouling properties.

Shibutani *et al.*^58^ reported that cellulose derivative membranes in which some of the acetyl groups of cellulose acetate were replaced with propionyl groups or butyryl groups resulted in significant fouling tendencies, compared with cellulose acetate membrane, when foulant-containing water is introduced. These findings consistent with aforementioned results obtained in our work that the lower the number of substituent carbon atoms, the higher the Δ*G*_132_ implying better the antifouling properties, verifying that the VCG theory is, to certain degree, useful in predicting antifouling properties of cellulose esters. For more comprehensive and/or practical predictions, a theory taking into account sort of ions, concentrations of ions, pH and the likes is required but we confine ourselves in the VCG theory in this work.

### 3.3 Cellulose esters with lauroyl and trioxadecanoyl (TOD) groups

When CA is used as a water treatment membrane, it has excellent antifouling properties, exhibits less decrease in permeation flux, and less increase in filtration pressure than hydrophobic membranes, such as PES and PVDF.^34^ Compared to hydrophobic membranes, such as PES, it can easily recover the permeation flow rate after washing.^59^ However, the CA membrane deteriorates and its strength decreases after washing with sodium hypochlorite solution, which is used to efficiently remove foulants.^41^ Furthermore, introducing bulky substituents such as benzoyl or stearoyl groups can prevent the deterioration of CA by washing it with sodium hypochlorite solution.^41^ Conversely, these bulky substituents have a large number of carbon atoms, which makes the polymer hydrophobic. Hydrophobic polymers have low antifouling property,^27^ and hence, introducing bulky and hydrophobic substituents may reduce the antifouling properties. One way to improve the antifouling properties of hydrophobic membranes is to introduce hydrophilic substituents.^60^ Therefore, we investigated the introduction of bulky (hydrophobic) substituents to improve the chlorine resistance of CA and the introduction of hydrophilic substituents to improve the antifouling properties, which were lowered by introducing hydrophobic substituents.

Benzoyl groups can be used to improve chlorine resistance.^41^ However, introducing benzoyl groups makes cellulose derivatives hard but brittle.^61^ If the polymer used for the membrane is brittle, it can break during membrane formation. Therefore, it is desirable that the polymer for the membrane has ductility (or tensile elongation) equal to or higher than that of CA. Furthermore, polymers with high *T*_g_ are desirable in terms of the ease of solidification during membrane formation. According to studies on the physical properties of cellulose fatty acid esters,^62,63^ ductility or tensile elongation increases and *T*_g_ decreases as the substituent carbon number of the cellulose triester approaches 7 or 8. Owing to the balance between ductility and *T*_g_, the lauroyl group was used to improve chlorine resistance in this study. Because the polyethylene oxide structure exhibits a large *γ*^−^ (ref. 46) and is expected to improve antifouling properties, the trioxadecanoyl (TOD) group, which has an oligoethylene oxide structure, is used as a hydrophilic substituent to improve the antifouling property.

In this study, three types of cellulose esters (CTA and CLTODs) with lauroyl and TOD groups were evaluated for their antifouling properties based on the VCG theory, chlorine resistance, and mechanical properties. The details of this process are described below.

To evaluate the interaction between cellulose derivatives and foulants using the VCG theory, we measured the contact angles of water, formamide, and diiodomethane on the surface of cellulose esters (CTL and CLTODs). For reference, the PES and CTA used in water treatment membranes were also measured. Table 7 presents the results. It was seen that the higher the DS of the TOD groups, the lower was the contact angle with water, and the TOD groups improved the surface hydrophilicity of the polymer. Table 8 shows the surface tension components of CTL, CLTODs, PES, and CTA(b), calculated from the contact angles shown in Table 7. The *γ*^−^ value of CTL was considerably smaller than that of CTA(b). Similar to the discussion of CTA to CTV in the previous section, the diluting effect of the substituents reduced *γ*^−^. The reason why the *γ*^−^ of CLTODs is larger than that of CTL is that the *γ*^−^ of the oligoethylene glycol structure of the TOD group was added to the *γ*^−^ of the cellulose ester moiety. The *γ*^+^ values of CTL and CLTODs were larger than those of PES and CTA, which were approximately 0. The reason is unknown.

**Table 7:** Average figures^a^, standard deviation, and the number of measurements of the measured contact angles of formamide, diiodomethane, and water on cellulose esters with long substituent (CTL and CLTODs), and PES and CTA for reference

Polymers	Formamide			Diiodomethane			Water		
	Average (deg)	SD (deg)	*n*	Average (deg)	SD (deg)	*n*	Average (deg)	SD (deg)	*n*
CTL	87.8^a^	1.1	10	54.3^a^	1.0	10	102.9^a^	0.4	10
CLTOD(a)	84.7^b^	1.5	10	46.0^b^	1.1	10	97.4^b^	0.4	10
CLTOD(b)	77.3^c^	1.6	10	47.5^b^	3.2	10	90.3^c^	0.8	10
PES	57.2^d^	2.6	10	28.0^d^	1.3	10	81.2^d^	1.3	10
CTA(b)	51.9^e^	1.8	10	36.2^e^	1.3	10	62.1^e^	1.0	10

a Values in the same column with different letters are significantly different (*p* < 0.05).

**Table 8:** Calculated surface tension components of cellulose esters with long substituent (CTL and CLTODs) and PES and CTA for reference

Polymers	*γ* ^LW^ (mJ m^−2^)	*γ* ^+^ (mJ m^−2^)	*γ* ^−^ (mJ m^−2^)
CTL	31.8	1.4	2.4
CLTOD(a)	36.5	2.2	4.7
CLTOD(b)	35.6	0.8	6.6
PES	45.0	0.007	4.8
CTA(b)	41.5	0.004	22.0

**Fig. 5:**
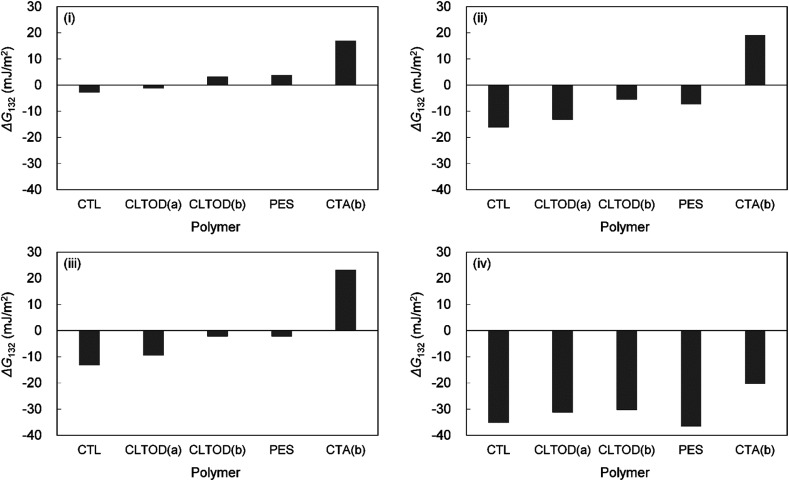
Calculated interfacial interaction energies (Δ*G*_132_) between foulants ((i) HSA, (ii) PEG, (iii) BSA, and (iv) HA) and cellulose esters with long substituent (CTL and CLTODs), as well as PES and CTA for reference.

Fig. 5 shows the free energy of the interfacial interaction (Δ*G*_132_) of the polymers (CTL, CLTODs, PES, and CTA(b)) and the foulants calculated from each surface tension component, as shown in Table 8. On comparing CTA(b) and CTL, it was found that CTL has a smaller Δ*G*_132_ than CTA(b) against any foulant, thereby indicating that CTL has lower fouling resistance than CTA. On comparing CTL and CLTODs, CLTOD(b), with the most TOD groups, had the largest Δ*G*_132_, and CTL with no TOD groups had the smallest Δ*G*_132_, thereby indicating that CLTOD(b) exhibited the best antifouling properties, and CTL exhibited worst antifouling properties in the three cellulose esters. Therefore, we can conclude that the TOD groups improved the antifouling properties of the polymer, as expected. However, the antifouling properties of CLTODs were still lower than those of CTA(b), and the antifouling property of CLTOD(b) was close to that of PES. The results of analyzing the breakdown of Δ*G*_132_ in the same manner as in the previous section are shown in Table 9. The following conclusions were drawn from these results. The absolute values of term (i) of CTL, CLTODs, PES, and CTA were small; therefore, the effects of *γ*^LW^ on Δ*G*_132_ were small. The *γ*^−^ values of CTL and CLTODs were small, so term (ii) of CTL and CLTODs was smaller than that of CTA(b). Because the *γ*^+^ of CTL and CLTODs was larger than that of CTA(b), term (iii) of CTL and CLTODs was less negative than that of CTA(b). For term (iv), the results differed depending on the foulant used. In summary, the antifouling properties of CTL and CLTODs were inferior to those of CTA because their *γ*^−^ values were smaller and *γ*^+^ of them were larger than that of CTA(b).

**Table 9:** Breakdown of the calculated interfacial interaction energies (Δ*G*_132_) between foulants (HSA, PEG, BSA, HA) and cellulose esters with long substituent (CTL and CLTODs), as well as PES and CTA for reference

Components of Δ*G*_132_		Foulants	CTL (mJ m^−2^)	CLTOD(a) (mJ m^−2^)	CLTOD(b) (mJ m^−2^)	CTA(b) (mJ m^−2^)	PES (mJ m^−2^)
(i)	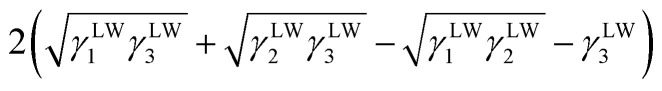	HSA	−1	−1	−1	−2	−2
		PEG	−4	−5	−5	−7	−8
		BSA	−1	−2	−2	−3	−3
		HA	−2	−2	−2	−3	−4
(ii)	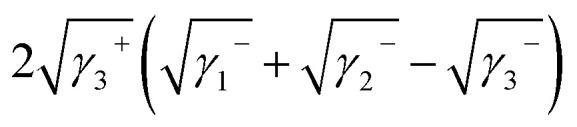	HSA	37	43	47	68	43
		PEG	46	52	56	77	52
		BSA	45	52	56	77	52
		HA	1	7	11	32	7
(iii)	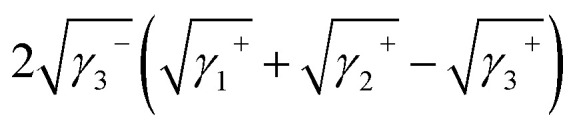	HSA	−14	−11	−16	−25	−25
		PEG	−39	−36	−42	−50	−50
		BSA	−39	−36	−42	−50	−50
		HA	−20	−17	−23	−31	−31
(iv)	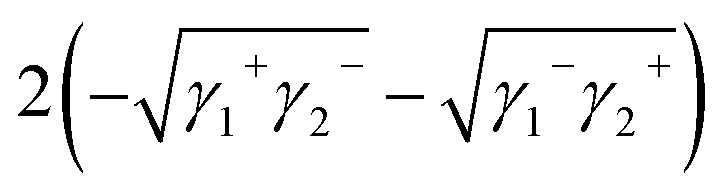	HSA	−25	−32	−26	−24	−12
		PEG	−19	−24	−15	−1	−1
		BSA	−19	−24	−15	−1	−1
		HA	−14	−19	−16	−18	−9
Δ*G*_132_	(i) + (ii) + (iii) + (iv)	HSA	−3	−1	3	17	4
		PEG	−16	−13	−6	19	−7
		BSA	−14	−10	−3	23	−3
		HA	−35	−31	−30	−20	−36

Because the *γ*^+^ of polyethylene oxide (PEG-6000) was 0 mJ m^−2^ and its *γ*^−^ was 58.5–64 mJ m^−2^ (ref. 46) and their values were better for the antifouling property than that of CLTODs, the length of the polyethylene oxide structure in the substituent should be increased to further improve the antifouling property of CLTOD, or the DS of the substituent with a polyethylene oxide structure should be increased.

The evaluation results of the chlorine resistance and equilibrium water content of CTL, CLTOD(a)(b), and PES and CTA(b) are shown in Table 10. The chlorine resistance of CTL with lauroyl groups was higher than that of CTA. CLTOD(a) with TOD groups (DS 1.3) exhibited higher chlorine resistance than CTL. Interestingly, CLTOD(b) with TOD groups (DS 1.9) had a lower chlorine resistance than CTL or CLTOD(a).

**Table 10:** Chlorine resistance and equilibrium moisture content measured on films of cellulose esters with long substituent (CTL and CLTODs) and PES and CTA for reference

Polymers	Chlorine resistance^a^ (h)	Equilibrium water content^b^ (%)
CTL	24–72	2.6
CLTOD(a)	>72	0.8
CLTOD(b)	<24	1.4
PES	>72	1.0
CTA(b)	<24	6.3

a Chlorine resistance is evaluated by measuring the period until the tensile strength decreased to 90%, using films immersed in an aqueous solution of sodium hypochlorite with an effective chlorine concentration of 20 000 ppm.

b Equilibrium water content is measured at 40 °C, 90% RH.

Additionally, the equilibrium water content of each polymer was measured. The equilibrium water content decreased in the order CTA(b), CTL, and CLTOD(a), which was the same as the order of chlorine resistance. If the equilibrium water content is low, that is, if the water in the polymer is low, the concentration of hypochlorite ions in the water in the polymer also is assumed to be low, leading to improved chlorine resistance. The equilibrium water content of CLTOD(b) was 1.4%, which was lower than that of CTL; however, its chlorine resistance was worse than that of CTL. CLTOD(b) had an equilibrium water content of 1.4%, which was lower than that of CTL; however, its chlorine resistance was worse than that of CTL. In the case of CLTOD(b), the relationship between chlorine resistance and equilibrium moisture content was different from that of the other cellulose derivatives. Although the reason for this is unclear, because the ether bonds of polyethylene oxide are oxidatively degraded by sodium hypochlorite,^64^ it is possible that CLTOD(b), which has many polyethylene oxide structures as TOD groups, was significantly affected by the decomposition of TOD groups, in addition to the effects of main-chain scission.

The mechanical property evaluation results for CTL, CLTOD, PES, and CTA(b) are shown in Table 11.

**Table 11:** Tensile strength, elongation at break, and tensile modulus measured on films of cellulose esters with long substituent (CTL and CLTODs), and PES and CTA for reference^a^

Polymers	Tensile strength (MPa)	Elongation at break (%)	Tensile modulus (MPa)
CTL	12	74	86
CLTOD(a)	11	146	67
CLTOD(b)	8	173	10
PES	64	6	1081
CTA(b)	97	12	1010

a Tensile tests are performed on films wetted with water.

Compared to the 12% elongation of CTA(b), the elongation of CTL, into which lauroyl groups were introduced, was improved to 74%. The CLTOD-containing TOD group showed better elongation than the CTL group. The elongations of CLTOD(a) and CLTOD(b) were 146% and 173%, respectively. The reason why the TOD group improved the elongation is that the water acted as a plasticizer on the hydrophilic TOD group. However, the introduction of the lauroyl and TOD groups significantly lowered the strength and elastic modulus compared to that of CTA(b). The improvement in this point is important when using long-chain acyl groups such as lauroyl groups and TOD groups in cellulose esters for water treatment of membrane materials.

### 3.4 Contact angle measurement with captive bubble method

The water contact angle of each polymer described thus far was measured by the sessile drop method using a dried film. However, because water treatment membranes are used in water, it is important to understand their physical properties and behavior in water. Because the contact angle of a polymer changes depending on the history of the substance it touches,^65^ it is desirable to evaluate the physical properties of the membrane in contact with water.^66^

Therefore, the contact angles of the polymers used in this study were measured in water using the captive bubble method^66,67^ and the contact angles in air were compared using the sessile drop method. Table 12 presents the results.

**Table 12:** Comparison between the sessile drop and captive bubble methods to determine the water contact angle^a^

Polymers	Sessile drop method			Captive bubble method			Difference
	Average (*A*) (deg)	SD (deg)	*n*	Average (*B*) (deg)	SD (deg)	*n*	*A* − *B* (deg)
CTL	102.9^a^	0.4	10	92.3^a^	1.0	10	10.6
CLTOD(a)	97.4^b^	0.4	10	59.7^b^	1.2	20	37.7
CLTOD(b)	90.3^c^	0.8	10	53.7^c^	0.7	20	36.6
PES	81.2^d^	1.3	10	67.6^d^	3.3	20	13.6
CTA(b)	62.1^e^	1.0	10	58.9^b^	2.7	39	3.2

a Values in the same column with different letters are significantly different (*p* < 0.05).

In the sessile drop method, the contact angles of CLTOD(a) and (b) were larger than those of PES and CTA. In other words, they were hydrophobic, although they had hydrophilic TOD groups. Conversely, using the captive bubble method, CLTOD with hydrophilic TOD groups was as hydrophilic as CTA. Additionally, CLTOD had a large difference (*A* − *B*) between the values of the captive bubble method and the sessile drop method, compared to CTA, PES, and CTL, considering the lauroyl and trioxadecanoyl groups, which are substituents of CLTOD, are flexible and easy to move, and the difference in hydrophilicity between these groups is large. This indicates that the hydrophobic lauroyl group can be exposed on the surface in air, whereas the hydrophilic trioxadecanoyl group tends to be exposed on the surface in water. In this study, we calculated the surface tension component from the contact angle based on VCG theory and evaluated the free energy of the interfacial interaction between the membrane and the foulant. Originally, the evaluation using the contact angle with the polymer in the hydrated state was considered closer to the evaluation of the actual system. However, further research is required in this regard.

## 4 Conclusion

Using VCG theory, this study evaluated the free energy at the interfaces between the cellulose esters and foulants, and the results were analyzed in detail. For the foulants with large *γ*^−^ and small *γ*^+^, polymers with large *γ*^−^ and small *γ*^+^, like the foulants, had large positive values of Δ*G*_132_ and were excellent in antifouling properties. Considering the size of the substituent on the cellulose triester, cellulose acetate, which had the smallest substituents, exhibited the best antifouling property, considering as the substituent increases, *γ*^−^ becomes more diluted by the volume of the larger substituent. Similarly, for a membrane with a large *γ*^−^ and small *γ*^+^, a foulant with a larger *γ*^−^ and smaller *γ*^+^ is less likely to adhere to the membrane. Therefore, analysis based on the VCG theory is a very effective tool that can quantitatively evaluate the adherence of foulants and membranes by simply measuring the contact angle.

On the other hand, pH, sort of ions, concentrations of ions and things like that are not what are dealt with by the VCG theory. In order for us to better understand the phenomena with a view to developing more useful membrane material, we certainly need to look to more sophisticated theory such as extended DLVO theory; this is an issue for future study.

Additionally, CLTOD with a lauroyl group was introduced to improve the chlorine resistance of cellulose, and a TOD group was introduced to improve the fouling resistance. The contact angle of CLTOD by the captive bubble method showed hydrophilicity equivalent to that of CTA; however, when evaluating the VCG theory, the antifouling property was inferior to that of CTA. Although CLTOD has higher chlorine resistance and ductility (tensile elongation at break) than CTA, it has lower strength and elastic modulus. To summarize, our research revealed that antifouling performance of cellulose esters decreases in general by increasing carbon number in substituent because of poor electron-donating nature of long aliphatic ester groups, and that, when a long aliphatic ester group is required in terms of other properties such as resistance to chlorine, introducing together another substituent with electron-donating nature such as ethylene glycol moiety could hit balance between antifouling and other performances.

## Conflicts of interest

The author(s) declare that there are no conflicts of interest to declare.

## Supplementary Material

## References

[cit1] Luo H., Aboki J., Ji Y. (2018). et al.. ACS Appl. Mater. Interfaces.

[cit2] Deshmukh A., Boo C., Karanikola V. (2018). et al.. Energy Environ. Sci..

[cit3] Sapkota B., Liang W., VahidMohammadi A. (2018). et al.. Nat. Commun..

[cit4] Liu J., Liu L., Huang Z. (2020). et al.. Pol. J. Environ. Stud..

[cit5] Rzasa M., Lukasiewicz E. (2021). Bull. Pol. Acad. Sci.: Tech. Sci..

[cit6] Newton E. H., Birkett J. D. (1969). Desalination.

[cit7] Membrane separation principles and applications: From material selection to mechanisms and industrial uses, ed. A. F. Ismal, M. A. Rahman, M. H. D. Othman and T. Matsuura, Elsevier, Amsterdam, 1st edn, 2018

[cit8] Yang F., Yan Z., Zhao J. (2020). et al.. J. Mater. Chem. A.

[cit9] Ahmed S. F., Mehejabin F., Momtahin A. (2022). et al.. Chemosphere.

[cit10] Guo H., Li X., Yang W., Yao Z. (2022). et al.. Front. Chem. Sci. Eng..

[cit11] Zhang J., Wu B., Zhang J., Zhai X. (2022). et al.. Water Res..

[cit12] Guo W., Ngo H. H., Li J. (2012). Bioresour. Technol..

[cit13] Guo Y., Li T. Y., Xiao K. (2020). et al.. J. Membr. Sci..

[cit14] Ujihara R., Mino Y., Takahashi T. (2016). et al.. J. Membr. Sci..

[cit15] Boulven E. Y., Triatmadja R., Kamulyan B. (2021). et al.. E3S Web Conf..

[cit16] Kuo D., Sakamoto T., Torii S. (2022). et al.. Polym. J..

[cit17] Colburn A. S., Meeks N., Weinman S. T. (2016). et al.. Ind. Eng. Chem. Res..

[cit18] Farhat N. M., Staal M., Bucs S. S. (2016). et al.. J. Membr. Sci..

[cit19] Gul A., Hruza J., Yalcinkaya F. (2021). Polymers.

[cit20] Ding J., Liang H., Zhu X. (2021). et al.. J. Membr. Sci..

[cit21] Nezam El-Din L. A., El-Gendi A., Ismail N., Abeda K. A., Ahmed A. I. (2015). J. Ind. Eng. Chem..

[cit22] Takao S., Rajabzadeh S., Otsubo C. (2022). et al.. ACS Omega.

[cit23] Zhao C., Xue J., Ran F., Sun S. (2013). Prog. Mater. Sci..

[cit24] Kucera J. (2019). Membranes.

[cit25] Kim I. C., Yun H. G., Lee K. H. (2002). J. Membr. Sci..

[cit26] Kang G., Cao Y. (2014). J. Membr. Sci..

[cit27] Diez B., Rosal R. (2020). Nanotechnol. Environ. Eng..

[cit28] Nunes S. P. (2020). Curr. Opin. Chem. Eng..

[cit29] Chen Y., Zhang J., Cohen Y. (2022). Sep. Purif. Technol..

[cit30] Abidin M. N. Z., Nasef M. M., Matsuura T. (2022). Polymers.

[cit31] Yan L., Yang X., Zhao Y., Wu Y. (2022). et al.. Sep. Purif. Technol..

[cit32] Barambu N. U., Bilad M. R., Wibisono Y. (2019). et al.. Polymers.

[cit33] Formoso P., Pantuso E. (2017). et al.. Membranes.

[cit34] Jeon S., Rajabzadeh S., Okamura R., Ishigami T., Hasegawa S., Kato N., Matsuyama H. (2016). Water.

[cit35] Rustemeyer P. (2004). Macromol. Symp..

[cit36] Okajima K., Kowsaka K., Kamide K. (1992). Polym. Int..

[cit37] Ho L. C. W., Martin D. D., Lindemann W. C. (1983). Appl. Environ. Microbiol..

[cit38] Loeb S. and Sourirajan S., Sea water demineralization by means of an osmotic membrane, in Saline water conversion—II (Advances in chemistry vol 38), American Chemical Society, 1963, pp.117–132

[cit39] Uemura T., Kurihara M. (2003). Bull. Soc. Sea Water Sci., Jpn..

[cit40] Nakatsuka S., Nakate I., Miyano T. (1996). Desalination.

[cit41] Hashizume T., Okamoto Y., Nagai K., Shimamoto S. (2022). Text. Res. J..

[cit42] Ohya H., Negishi Y., Matsui K. (1981). et al.. Kagaku Kogaku Ronbunshu.

[cit43] Ohya H., Negishi Y., Matsui K. (1981). et al.. Kagaku Kogaku Ronbunshu.

[cit44] Yuan W., Zydney A. L. (1999). J. Membr. Sci..

[cit45] Her N., Amy G., Plottu-Pecheux A., Yoon Y. (2007). Water Res..

[cit46] van Oss C. J. , Interfacial Forces in Aqueous media, Taylor & Francis, Boca Raton, 2nd edn, 2006

[cit47] van Oss C. J., Chaudhury M. K., Good R. J. (1988). Chem. Rev..

[cit48] Cornelissen E. R., van den Boomgaard T., Strathmann H. (1998). Colloids Surf., A.

[cit49] Białopiotrowicz T., Jańczuk B. (2002). Appl. Surf. Sci..

[cit50] Subhi N., Verliefde A. R. D., Chen V., Le-Clech P. (2012). J. Membr. Sci..

[cit51] Meng X., Luosang D., Meng S. (2021). et al.. Chemosphere.

[cit52] Ma Y., Zydney A. L., Chew J. W. (2021). AIChE J..

[cit53] Shibutani T., Kitaura T., Ohmukai Y. (2011). et al.. J. Membr. Sci..

[cit54] Röder T., Moosbauer J., Kliba G. (2009). et al.. Lenzinger Ber..

[cit55] Malm C. J., Genung L. G., Fleckenstein J. V. (1947). Ind. Eng. Chem..

[cit56] Suarez F., Romero C. M. (2011). J. Chem. Eng. Data.

[cit57] Meng X., Tang W. (2015). et al.. J. Membr. Sci..

[cit58] Shibutani T., Kitaura T. (2011). et al.. J. Membr. Sci..

[cit59] Nakatsuka S., Watabe T. (2014). Membrane.

[cit60] Van der Bruggen B. (2009). J. Appl. Polym. Sci..

[cit61] Braun D., Bahlig K. H. (1994). Die Angewandte Makromolekulare Chemie.

[cit62] Danjo T., Iwata T. (2018). Polymer.

[cit63] Duchatel-Crépya L., Jolyb N., Martinb P. (2011). et al.. Carbohydr. Polym..

[cit64] Ogata Y., Kimura M. (1979). Yuki Gosei Kagaku Kyokaishi.

[cit65] Grundke K., Pöschel K., Synytska A. (2015). et al.. Adv. Colloid Interface Sci..

[cit66] Baek Y., Kang J., Yoon P. J. (2012). Desalination.

[cit67] Prydatko A. V., Belyaeva L. A., Jiang L., Lima L. M. C., Schneider G. F. (2018). Nat. Commun..

